# Intrauterine growth restriction alters nutrient metabolism in the intestine of porcine offspring

**DOI:** 10.1186/s40104-020-00538-y

**Published:** 2021-02-08

**Authors:** Tiantian Li, Shimeng Huang, Long Lei, Shiyu Tao, Yi Xiong, Guoyao Wu, Jie Hu, Xiongkun Yuan, Shengjun Zhao, Bin Zuo, Hongjian Yang, Yingping Xiao, Gang Lin, Junjun Wang

**Affiliations:** 1 State Key Laboratory of Animal Nutrition, College of Animal Science and Technology, China Agricultural University, Beijing, 100193 People’s Republic of China; 2 Hubei Collaborative Innovation Center for Animal Nutrition and Feed Safety, Hubei Key Laboratory of Animal Nutrition and Feed Science, Wuhan Polytechnic University, Wuhan, 430023 People’s Republic of China; 3 Department of Animal Science, Texas A&M University, College Station, TX 77843 USA; 4 State Key Laboratory for Managing Biotic and Chemical Threats to the Quality and Safety of Agro-products, Institute of Quality and Standard for Agro-products, Zhejiang Academy of Agricultural Sciences, Hangzhou, 310021 People’s Republic of China; 5 Key Laboratory of Agrifood Safety and Quality, Institute of Quality Standards and Testing Technology for Agricultural Products, Chinese Academy of Agricultural Sciences, Ministry of Agriculture and Rural Affairs, Beijing, 100081 People’s Republic of China

**Keywords:** Absorption, Digestion, Fermentation, IUGR pigs, Portal vein

## Abstract

**Background:**

Intrauterine growth restriction (IUGR) has negative impacts on the postnatal survival, growth and development of humans and animals, with not only on newborns but also adulthood. However, the characteristics for nutrient digestion and absorption in IUGR offspring are still largely unknown. Therefore, the normal birth weight (NBW) and IUGR growing pigs were used in this study to investigate their differences in nutrient utilization, with an expectition for further nutritional optimization of the IUGR offspring during their later life.

**Methods:**

Twelve IUGR and 12 NBW growing pigs were fitted with catheters in their portal vein to measure blood flow rate as well as nutrients and metabolites in plasma. The digestibilities of nutrients in different intestinal segments, and bacterial fermentation in the large intestine were examined to reveal the characteristics of nutrients utilization in IUGR versus NBW pigs.

**Results:**

The rate of portal venous blood flow did not differ beween IUGR and NBW pigs. Plasma concentrations of total cholesterol, triglycerides and glucose were much lower but those of urea were higher in the portal vein of IUGR pigs, compared with the NBW pigs. The ileal digestibility of dry matter, gross energy and starch were lower in IUGR pigs than in NBW pigs. IUGR increased hindgut microbial diversity and bacterial fermentation activity in the caecum. *In vitro* cross-fermentation of ileal digesta by caecal microbes of NBW and IUGR pigs showed that gas production was much higher for IUGR ileal digesta regardless of the source of caecal inocula.

**Conclusion:**

IUGR impairs the nutrient digestion and absorption in small intestine, reduces caecal microbial diversity and promotes bacterial fermentation in the large intestine during the growing phase. These findings aid in our understanding of nutrient metabolism in IUGR pigs and provide the basis for future nutritional interventions.

## Background

Intrauterine growth restriction (IUGR) is defined as impaired growth and development of the mammalian embryo/fetus or its organs during pregnancy, which can be measured as fetal or birth weight less than two standard deviation of the mean body weight for gestational age [1, 2]. Despite advanced prenatal care for both mothers and fetuses, approximately 15% of human infants suffer from IUGR worldwide [3]. Evidences suggest that IUGR negatively impacts not only newborns but also their postnatal growth and development into adulthood [4]. Previous studies have shown that young adults with IUGR have a higher probability of developing organ dysfunction than those born with normal birth weight [5, 6]. Efficient use of nutrients by the gastrointestinal tract is a prerequisite for ensuring health of the body. Therefore, exploring the metabolic patterns of IUGR individuals in adulthood is conducive to a deeper understanding of the impact of the IUGR on the life cycle of humans and animals.

The small intestine is the major organ for terminal digestion and absorption of dietary nutrients [7]. Proteins and digestible carbohydrates (including starch and disaccharides) are rapidly digested and the resultant products absorbed by the small intestine [7, 8]. However, in nonruminants, a large quantity of undigested dietary carbohydrates passes into the large intestine where they are fermented by bacteria to produce short-chain fatty acids [9]. The digested nutrients and fermented products are absorbed into the portal vein for utilization by animals, which can be analyzed and quantified as an important target in nutritional research [10]. Recent study in our laboratory has shown that IUGR compromizes gut epithelial barrier function in growing pigs [11]. Therefore, we hypothesized that IUGR might negatively affect the digestion and absorption of nutrients in small intestine and influence microbial fermentation in the hindgut.

The pig exhibits the most severe naturally occurring IUGR among mammals, with IUGR piglets accounting for about 15–20% in newborn piglets [12]. The growth phase of humans and animals is the golden age of the entire life process. Due to similarities in digestion and metabolism between pigs and humans, researchers usually choose the pigs as a model to study the gastrointestinal physiology and function of humans with IUGR. The purpose of this study was to identify the effect of IUGR on nutrient metabolism in the whole intestine of IUGR pigs during the growth phase.

## Methods

### Animals, surgery and sample collection

A batch of 289 Landrace × Yorkshire newborns was weighted immediately after birth at the Ministry of Agriculture Feed Industry Center Animal Testing Base (Hebei, China). The sows with very similar parities (3–4) were selected and the semen of same origin was used when proceeding with artificial insemination. These newborns were marked and further confirmed to be used for experiments. Among them, 12 IUGR and 12 normal birth weight (NBW) barrows were finally selected at d 85 of age. These piglets were selected based on the IUGR identification method [13] and then their littermate NBW pigs were selected correspondingly as well as healthy status. The average birth weights of IUGR and NBW pigs were 0.9 ± 0.14 kg and 1.4 ± 0.15 kg, respectively.

The pigs were individually placed into stainless-steel metabolism cages (1.4 m × 0.45 m × 0.6 m) in the Laboratory of Animal Metabolism in Research Unit of China Agricultural University (Beijing, China). The pigs were weighted before the surgery and the average body weights of IUGR and NBW pigs were 29 ± 1.8 kg and 36 ± 2.1 kg, respectively. Chronic catheters were then surgically placed into the hepatic portal vein, cranial mesenteric vein, and femoral artery following the surgery procedure reported previously [10]. The catheter was blocked with a heparin lock and the pig was given IM injection of antibiotic (cefazolin) at 50 mg/kg. All pigs were adapted to the cages and the catheters for 7 d prior to the start of the experiment, and fed the same diet twice daily at 08:00 and 17:00 with a feed intake based on their body weight (4% of initial body weight) for another 7 d before the collection of blood sample. The detailed description of diet was described at our previous study [14]. Feed refusals and spillage were recorded daily. Water was freely available from low-pressure drinking nipples. During the sampling collection, morning feed was equally divided into 6 parts and fed to pigs every hour to obtain metabolic changes [14]. Preliminary experiments were done to ensure that pigs could consume all the feed provided within a certain time period.

### Blood sample collection and flow rate measurement

Baseline samples were collected 10 min before morning feeding and then blood samples were collected through fitted catheters every hour after feeding. The flow rate of blood in the portal vein was estimated by the indicator-dilution technique and *p*-amino hippurate (PAH) was used as the indicator and infused through the cranial mesenteric vein catheter. The blood flow, portal vein plasma flow (PVPF), was calculated by equation: PVPF = Ci × IR / (PAHpv - PAHa) in which Ci is the concentration of PAH solution (mg/mL), IR is PAH infusion rate (mL/min); PAHpv and PAHa represent the concentrations of PAH (mg/mL) in portal vein and femoral arterial, respectively [15, 16]. The total nutrient flow was calculated through multiply the nutrient concentrations by blood flow rate. After finishing all the planned sampling, the pigs were killed to collect all the digesta of the jejunum, ileum, caecum and colon.

### Measurement of metabolites and biochemical parameters in the portal vein

Plasma metabolites were extracted by using 800 μL of ice-cold extraction mix (acetonitrile:methanol=1:1 v/v) in a 1:4 (sample: extraction solution) ratio. After 5-min vortex, the samples were centrifuged at 18,000×*g* for 10 min at 4 °C. Then, the supernatant fractions were collected and evaporated to dryness using a vacuum concentrator (Concentrator plus, Eppendorf). The resultant dry residues were re-suspended in 200 μL of 50% methanol, vortex-mixed and centrifuged again at 18,000 × *g* for 10 min at 4 °C. At last, the supernatant fractions were filtered through a 0.1-μm membrane and transferred to sampler vials to be analyzed on the LC-MS system. All the biochemical parameters were analyzed using Hitachi 7020 Chemistry Analyzer according to Wang et al. [17]. The absorption of nutrients (triglyceride, glucose, total cholesterol, plasma calcium, albumin, high-density lipoprotein, low-density lipoprotein and plasma urea nitrogen) into the portal vein of NBW and IUGR pigs were calculated based on blood flow and nutrient concentrations.

### Nutrient digestibility

Feces, digesta, and diet were analyzed for dry matter (DM), crude protein (CP) and gross energy (GE) [18]. All chemical analyses were conducted in duplicate. Inherent acid-insoluble ash (AIA) were measured and used as internal markers to estimate the nutrient digestibility at each intestinal site (ileum, caecum and colon) [19]. Starch was measured by the enzymic method [20] and starch digestibility was determined based on the report of Sun et al. [21].

### Bacterial fermentation activity

The concentrations of SCFAs in digesta from the caecum and colon were determined as previously described with slight modifications. Briefly, 0.5 g of digesta samples were weighed, diluted with 8 mL ultrapure water, homogenized and then centrifuged at 10,000×*g* for 20 min at 4 °C. The supernatant fluid was kept in a 2-mL screw-cap vial. Formic, acetic, propionic, butyric, and lactic acid were measured with a Dionex ICS-3000 Ion Chromatography System (Dionex Corporation, Sunnyvale, CA, USA) [22], whereas isobutyric, valeric, and isovaleric acid were measured by gas chromatography [23]. The calculation of SCFAs concentrations was based on the analyzed dry matter of the digesta.

### Gut microbiota sequencing

Microbial DNA was extracted from digesta using a QIAamp DNA Stool Mini Kit (Qiagen, GmbH Hilden, Germany) with an addition of a bead-beating step. Successful DNA isolation was confirmed by agarose gel electrophoresis. The V3-V4 region of the 16S rRNA gene was amplified with primer 341F (5′-CCTAYGGGRBGCASCAG-3′) and the reverse primer 806R (5′-GGACTACNNGGGTATCTAAT-3′). The optimized conditions for PCR amplification were as follows: initial denaturation at 95 °C for 5 min and 27 cycles of denaturation at 95 °C for 30 s, annealing at 55 °C for 30 s, and elongation at 72 °C for 45 s, followed by a final extension at 72 °C for 10 min. The resulting amplicons were gel purified, quantified, pooled and sequenced on the Illumina HiSeq 2500 platform. Sequence reads were processed through QIIME 1.8 (QIIME Team). After quality filtering, the sequences were denoised using denoise_wrapper.py. Denoised sequences were clustered into operational taxonomic units (OTUs) at a 97% sequence identify against the Greengenes Database (gg_13_8_otus). The chimeric OTUs were removed using UCHIME v4.2. Representative sequences for each OTU were picked and aligned using QIIME 1.8. Taxon-dependent analysis was conducted using the Ribosomal Database Project (RDP) classifier. OTUs were counted for each sample to express the richness of bacterial species with an identity cutoff of 97%. Alpha and beta diversity calculations and taxonomic community assessments were performed using QIIME 1.8 scripts.

### *In vitro* fermentation

The ileal digesta of every NBW and IUGR pigs was fermented by the caecal inocula from both NBW and IUGR pigs to verify the differences of fermentation activity in the caecum of NBW and IUGR pigs. The incubation system was the same as the previous study [24]. Briefly, the ileal digesta was dried by vacuum freeze-drying machine and sterilized by Cobalt-60 irradiation sterilization. Then the digesta was put into a sterile glass bottle and dissolved in a buffer solution waiting for fermentation. The caecal digesta was dissolved in the normal saline with a proportion of 1:10 and, and the mixture was centrifuged 10 min to collect the supernatant as the inocula. Glass bottles containing 0.5 mg digesta, 5 mL inocula, and 77 mL buffer solution were individually connected with a medical infusion pipe to the gas inlet of an automated gas recording system (AGRS-III, China Agricultural University, Beijing) to continuously record cumulative gas production. Each treatment contained four replicates. The microbial fermentation at 39 °C lasted for 24 h, and biomass culture fluids were collected for SCFA quantification.

### Statistical analysis

Statistical analyses were performed using SAS (version 9.2). For normally distributed continuous variables, the mean values were examined using the unpaired Student’s *t*-test. The level of significance was set at *P* <  0.05. The microbial data were analysed on the online platform of Majorbio I-Sanger Cloud Platform. Pathway analysis of metabolite profiles was carried out using MetaboAnalyst 3.0 (http://www.metaboanalyst.ca). Correlations between SCFAs concentrations (or nutrient digestibility) and relative abundance of bacterial taxa at genus level were tested with Spearman’s correlation and visualised using Canoco5 (Microcomputer Power, Ithaca, NY, USA).

## Results

### Differences in nutrient absorption into the portal vein between NBW and IUGR pigs

The rate of portal venous blood flow did not differ (*P* > 0.05) beween IUGR and NBW pigs (Table 1). However, plasma concentrations of total cholesterol, triglycerides and glucose were lower (*P* <  0.05) but those of urea were higher (*P* <  0.05) in the portal vein of IUGR pigs, compared with NBW pigs. Plasma concentrations of ALB, HDL, or LDL did not differ between NBW and IUGR pigs.

**Table 1:** Differential plasma metabolites and blood flow in portal vein of NBW and IUGR pigs^*^

Index	NBW	IUGR	***P-***value
Total cholesterol, mmol/L	2.01 ± 0.13	1.52 ± 0.05	0.01
Triglyceride, mmol/L	0.48 ± 0.06^a^	0.29 ± 0.06^b^	< 0.001
Urea nitrogen, mmol/L	3.25 ± 0.46	3.82 ± 0.15	0.04
Glucose, mmol/L	8.01 ± 0.72	7.04 ± 0.68	0.02
Albumin, g/L	33.88 ± 1.98	31.95 ± 2.87	0.76
High-density lipoprotein, mmol/L	0.39 ± 0.07	0.42 ± 0.13	0.79
Low-density lipoprotein, mmol/L	1.37 ± 0.26	1.36 ± 0.39	0.88
PVBF, mL/(min·kg BW)	55.75 ± 9.02	59.24 ± 11.09	0.63

*NBW normal birth weight, *IUGR* intrauterine growth restriction. Values are means ± SEM, *n*=12. Means with different lowercase letters represent differences between NBW and IUGR pigs

**Fig. 1:**
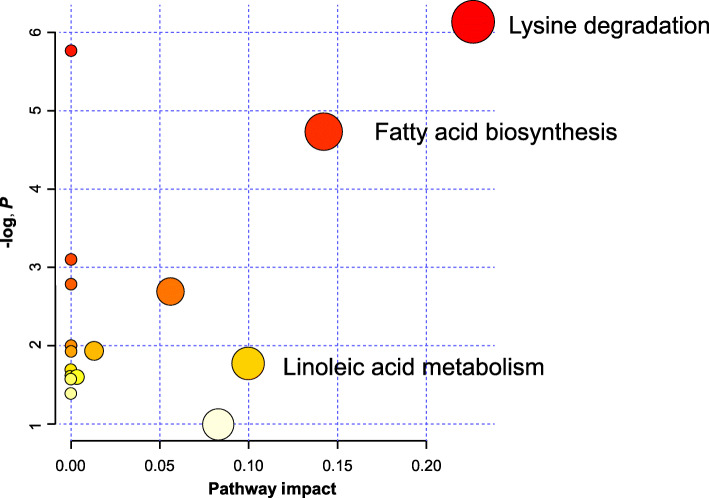
Pathway distribution of differential metabolites in the portal vein of NBW and IUGR pigs. Differential metabolites between NBW and IUGR pigs were used to analyzes the pathways. Node radius size and importance (X axis) reflects the pathway impact values calculated using betweenness centrality, which takes into consideration the global network structure and measures the number of shortest paths going through metabolites within the node. Node color and direction (Y axis), however, is based on the calculated *P* value of the enrichment analysis. Labeled pathways were up-regulated in IUGR pigs. NBW, normal birth weight; IUGR, low birth weight

Results of metabolomics study showed that the plasma concentrations of most of amino acids and pipecolic acid in the portal vein were lower (*P* <  0.05) but 5-aminopentanoic acid were higher (*P* <  0.05) in IUGR pigs than in NBW pigs (Table 2). Plasma concentrations of lysine metabolites were higher (*P* <  0.05), while those of fatty acid metabolites were lower (*P* <  0.05) in the portal vein of IUGR pigs (Fig. 1).

**Table 2:** Identified metabolites with significant differences between NBW and IUGR pigs^a^

No.	Description	MS ^**b**^	Formula	Fold change ^**c**^	*P*-value
1	Proline	115.1307	C_5_H_9_NO_2_	1.40	< 0.05
2	Arginine	175.0701	C_6_H_14_N_4_O_2_	1.82	< 0.05
3	Valine	117.0812	C_5_H_11_NO_2_	1.32	< 0.05
4	Ornithine	133.1601	C_5_H_12_N_2_O_2_	2.29	< 0.05
5	Threonine	120.0644	C_4_H_9_NO_3_	2.31	< 0.05
6	5-Aminopentanoic acid	117.1513	C_5_H_11_NO_2_	0.46	< 0.05
7	Lysine	147.1305	C_6_H_14_N_2_O_2_	0.79	< 0.05
8	Pipecolic acid	129.1357	C_6_H_11_NO_2_	0.76	< 0.05
9	Palmitelaidic acid	254.2382	C_16_H_30_O_2_	2.11	< 0.05
10	Oleic acid	282.7423	C_18_H_34_O_2_	1.65	< 0.05

^a^*NBW* normal birth weight, *IUGR* intrauterine growth restriction, *n* = 12

^b^Actual M-to-Z ratio

^c^Fold change was calculated by dividing the mean of normalized intensity of each plasma metabolite in the former by the mean of normalized intensity of each plasma in the latter. Fold change > 1 indicates that the metabolite was up-regulated in NBW pigs, whereas fold change < 1 indicates the metabolite was down-regulated

### Differences in nutrient digestibility between NBW and IUGR pigs in different intestinal segments

The digestibilities of DM, GE and starch were lower (*P* <  0.05) in the ileum of IUGR pigs than in NBW pigs (Table 3). In contrast, there was no difference (*P* > 0.05) in the digestibilities of nutrients in the colon, or the total tract between these two groups of pigs. DM and GE digestibilities in the caecum were higher (*P* <  0.05) in IUGR pigs than in NBW pigs.

**Table 3:** Ileal digestibility of nutrients in NBW and IUGR pigs*

Index	NBW	IUGR	*P*- value
DM, %	73.5 ± 0.6^a^	72.1 ± 0.6^b^	0.02
GE, %	79.3 ± 1.2^a^	76.5 ± 0.9^b^	0.01
CP, %	79.8 ± 1.4	78.4 ± 1.5	0.23
Starch, %	96.7 ± 0.8^a^	92.6 ± 0.7^b^	< 0.01

*NBW normal birth weight, *IUGR* intrauterine growth restriction. Values are means ± SE, *n* = 12. Means with different lowercase letters represent differences between NBW and IUGR pigs

### Differences in cecal or colonic microbial fermentation activity between NBW and IUGR pigs

To determine the microbial fermentation activity in the caecum and colon of NBW and IUGR pigs, the concentration of acetate, propionate, and butyrate in the digesta of the corresponding intestinal segments were measured. The results showed that the production of acetate, propionate and butyrate was 17%, 23% and 17% higher, respectively, in the caecum of IUGR than in NBW pigs (*P* <  0.05) (Table 4). In the colon, propionate production was 17% higher (Table 4) in IUGR than in NBW pigs (*P* <  0.05). The production of acetate and butyrate in the colon did not differ (*P* > 0.05) between the two groups of pigs.

**Table 4:** Concentrations of SCFAs in the caecal and colonic digestae of NBW and IUGR pigs^a^

Index	Cecum			Cecum		
	NBW	IUGR	*P*- value	NBW	IUGR	*P*- value
Acetate, mg/kg	3767.4 ± 378.0	4414.4 ± 371.5	0.02	4340.1 ± 473.1	4574.2 ± 342.3	0.65
Propionate, mg/kg	2529.4 ± 203.8	3116.8 ± 197.1	0.01	2142.8 ± 171.0	2500.5 ± 160.0	0.03
Butyrate, mg/kg	1153.2 ± 229.6	1346.1 ± 162.0	0.04	1351.7 ± 186.8	1287.4 ± 161.4	0.52

^a^*NBW* normal birth weight, *IUGR* intrauterine growth restriction. Values are means ± SE, *n* = 12

### Profiles of the microbial community in the caecum and colon of NBW and IUGR pigs

**Fig. 2:**
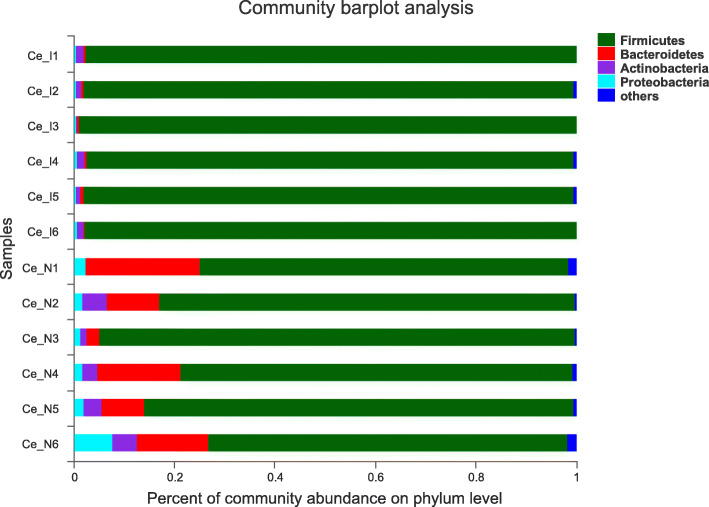
Microbiota composition in the caecal digestae of NBW and IUGR pigs (phylum level). NBW, normal birth weight; IUGR, low birth weight. Ce_I, caecal digestae of IUGR pigs; Ce_N, caecal digestae of NBW pigs. *n* = 6

**Fig. 3:**
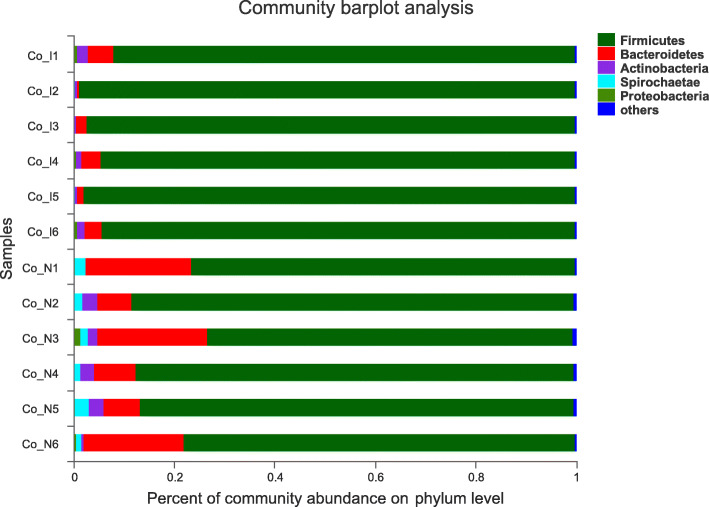
Microbial composition in colonic digestae of NBW and IUGR pigs (phylum level). NBW, normal birth weight; IUGR, low birth weight. Co_I, colonic digestae of IUGR pigs; Co_N, colonic digestae of NBW pigs. *n* = 6

Caecal or colonic microbial structure differed between NBW and IUGR pigs. Specifically, the microbial community of IUGR pigs had a lower abundance and less diversity in both caecum and colon (*P* <  0.05). Compared with NBW pigs, Firmicutes were more dominant (97% greater in the caecum and 92% greater in the colon) but Bacteroidetes were less abundant (13% greater in the caecum and 15% greater in the colon) in IUGR growing pigs (Fig. 2 and Fig. 3).

**Fig. 4:**
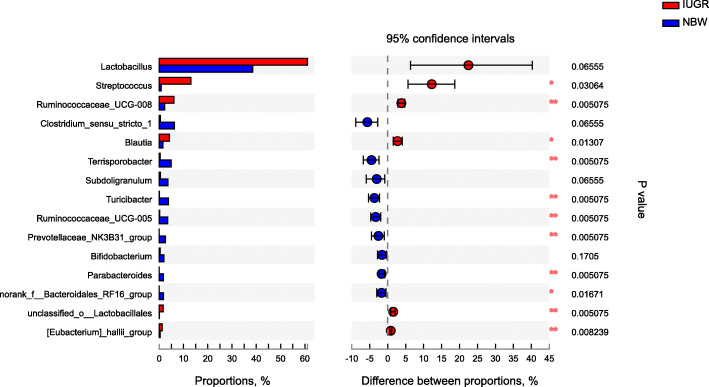
Differential bacterial genera in caecal digestae between NBW and IUGR pigs. Wilcoxon rank sum tes on genus level. NBW, normal birth weight; IUGR, low birth weight. *n* = 6

**Fig. 5:**
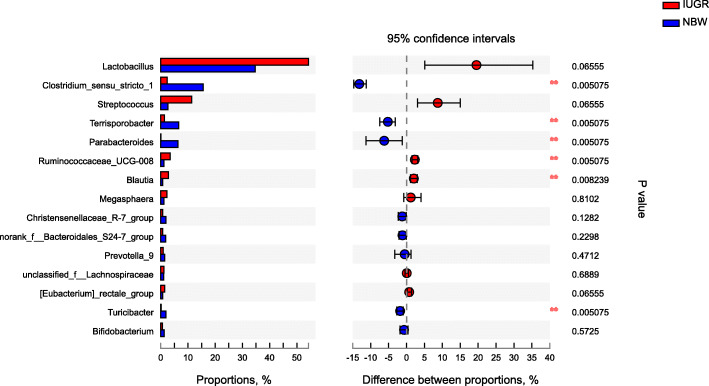
Differential bacterial genera in colonic digestae between NBW and IUGR pigs. Wilcoxon rank sum tes on genus level. NBW, normal birth weight; IUGR, low birth weight. *n* = 6

At the genus level, *Lactobacillus* was most abundant bacterial genera in the caecum and colon of NBW and IUGR pigs. *Streptococcus, Ruminococcaceae_UCG-008* and *Blautia* were more abundant (*P* <  0.05) while *Clostridium_sensu_stricro_1, Terrisporobacter,* and *Parabacteroides* were less abundant (*P* <  0.05) in the caecum and colon of IUGR pigs, compared with NBW pigs (Fig. 4 and Fig. 5).

### Correlation network analysis of shifts in microbiota composition, nutrient digestibility and production of SCFAs

The correlation network analysis was performed to investigate the potential links among microbiota, nutrient digestibility and SCFA production. The results showed that in the caecum, *Lachnoclostridium, Enterorhabdus, Anaerofustis* and *Bacteroides* were positively correlated with dry matter digestibility, but *Ruminococcus_1* and dominant *Latobocillus* was negatively correlated with dry matter digestibility (Figs. 7 and 8).

A greater number of bacterial genera were significantly correlated with acetate, such as *Lachnospiraceae_UCG-010*, *Faecalibacterium* and *Prevotella_9*. *Ruminococcaceae* was also positively correlated with acetate but negatively correlated butyrate concentrations in the hindgut of IUGR pigs. *Paraeggerthell* was positively correlated with butyrate. *Kitasatospora* and *Prevotella_2* was negatively correlated propionate. No bacterial genera were significantly correlated with acetate, propionate and butyrate at the same time.

### *In vitro* cross-fermentation of ileal digesta by caecal microbes from NBW and IUGR pigs for verification of relationships between microbes and nutrient digestibility

**Fig. 6:**
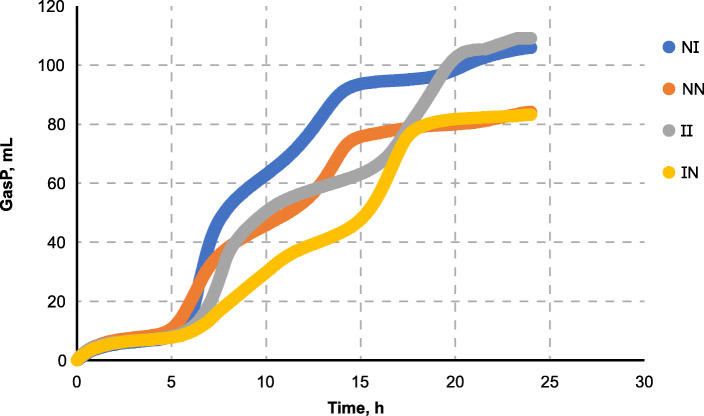
*In vitro* cumulative gas production during the cross-fermentation of ileal digestae from normal birth weight (NBW) or intrauterine growth restriction (IUGR) pigs by their caecal microbes. NI (NBW microbes plus IUGR digesta), NN (NBW microbes plus NBW digesta), II (IUGR microbes plus IUGR digesta), IN (IUGR microbes plus NBW digesta)

To determine whether higher level fermentation in the caecum of IUGR were caused by undigested nutrients and altered microbial composition in the caecum, we conducted an *in vitro* fermentation experiment. The real-time gas production monitoring system showed that the rate of microbial fermentation increased quickly with ileal digesta from IUGR pigs and gas production was much higher when ileal digestae from IUGR pigs was used as substrates regardless of the source of caecal inocula (Table 5). Accordingly, *in vitro* microbial fermentation capacity of IUGR digesta was higher than that in NBW pigs (Fig. 6).

**Table 5:** *In vitro* gas production during the fermentation of ileal digesta by caecal microbes from NBW and IUGR pigs^a^

	NBW digesta, mL	IUGR digesta, mL	***P***-value
NBW Inocula	86.3 ± 3.0	101.3 ± 12.0	0.01
IUGR Inocula	81.5 ± 9.0	111.3 ± 15.1	0.004

^a^*NBW* normal birth weight, *IUGR* intrauterine growth restriction. Values are means ± SE, n = 12

Gas production in the presence of the caecal inocula from IUGR pigs increased more quickly during the first 15 h, compared with the caecal inocula from NBW pigs.

## Discussion

IUGR infants consistently suffer from the impairment of organ development and susceptibility to infection and disease, resulting in high rates of postnatal mortality and reduced growth rates. But differences in nutrient utilization between NBW and IUGR growing pigs were largely unknown. In the current study, we used the growing pigs as a model to study the metabolic utilization of nutrients in the whole intestine. We found that IUGR growing pigs had defects in nutrient absorption by the small intestine into the portal vein. In addition, caecal microbial fermentation was higher in IUGR pigs than in NBW pigs, indicating that the higher-level fermentation was mainly caused by a great amount of the undigested nutrients (e.g., starch) from the small intestine. This further reduced the diversity of caecal microbiota in IUGR pigs. Further correlation analysis showed a complex interaction among digestion, absorption and microbial fermentation.

The small intestine is an important site for carbohydrate and protein digestion. Our results showed that the digestive function of the IUGR intestine was influenced by IUGR in a segment-dependent manner. Specifically, the ileal digestibilities of nutrients was higher in IUGR than in NBW pigs, but total tract digestibilities of nutrients did not differ between the two groups of pigs. Interestingly, the microbial composition of the caecum or colon were different between NBW and IUGR pigs, and this may result from the presence of different substrates in the colon of IUGR pigs, compared with NBW pigs. Results of our previous study with newborn pigs indicated that IUGR negatively affects expression of proteins involved in key biological processes such as absorption, digestion and transport of nutrients, and protein synthesis in the small intestine of newborn and preweaning piglets [25, 26]. It is plausible that IUGR pigs had a lower digestive capacity, a lower survival rate and a lower rate of weight gain due to the continuous impairment in the development of the small intestine from birth to weaning [26, 27]. However, to our surprise, the rate of fermentation was higher in the cecum of IUGR pigs, compared with their NBW littermates. There are reports that the hindgut fermentation for the production of SCFAs can provide energy from indigestible nutrients when the fatty acids are oxidized by colonocytes and good for enhancing growth [28–30]. Our recent study also indicated that pigs with a higher feed efficiency had a higher abundance of SCFA-producing bacteria [31]. Pigs that showed high level caecal fermentation may obtain up to 30% of their energy requirement for maintenance from microbially produced SCFAs in the large intestine [32]. The substrates were likely the starch that escaped the small intestine. In this way, the fermentation is a waste rather than a contribution to energy supply. On the other hand, acetic acid is the predominant SCFA in the colon [33]. After pigs consume a high fiber diet, their large intestine produced most acetic acid (52%), followed by propionic and butyric acids (36 and 8.5%, respectively) [33]. The differences in SCFA concentrations between NBW and IUGR pigs can be attributed to an altered microbial community and different digestibilities in the large intestine.

Some of the differences in digestion were also shown at the absorption level. Digestion and absorption of GE are directly associated. IUGR pigs had a lower ability to digest dietary energy and starch, resulting in a lower concentration of glucose in the portal vein. Hepatic glucose production can be regulated by insulin concentration in the portal vein [34]. However, when the digestibility of starch is reduced, the absorption of energy substrates into the portal vein will decline [35]. Thus, reduced digestion can decrease the rate of glucose absorption and the concentration of insulin in the portal vein [36]. Although there was no difference in protein digestibility between IUGR and NBW pigs, plasma urea concentration in the portal vein was higher in IUGR than in NBW pigs. As an indicator of nitrogen metabolism [37], a higher level of plasma urea concentration may result from increased catabolism of amino acids possibly due to their imbalance or a deficiency in one or more amino acids. The concentrations of total cholesterol and triglycerides in the portal vein were were much lower in IUGR pigs than in NBW pigs, possibly due to reduced absorption of fatty acids by the small intestine. Accumulation of fatty acids in plasma is regulated by many factors, including dietary intake of lipids, triglyceride hydrolysis, and fatty acid oxidation [38]. Fat digestibility was not measured in the current study, but fatty acid absorption appeared to be reduced in IUGR pigs compared with NBW pigs.

**Fig. 7:**
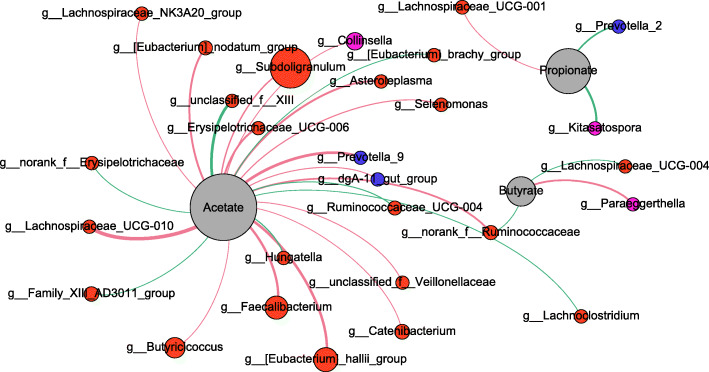
Correlation network analysis between short-chain fatty acid (SCFA) levels and the microbiota of IUGR and NBW pigs. Networks display Spearman’s correlations between the relative abundance of bacterial genus and SCFAs concentrations. Only correlations with corrected *P* value < 0.05 are illustrated. Nodes are colored according to phylum, and the size is proportional to the mean relative abundance within the population. Blue edges represent negative correlation, while pink edges represent positive correlation. The thickness of edges is proportional to the correlation coefficient

**Fig. 8:**
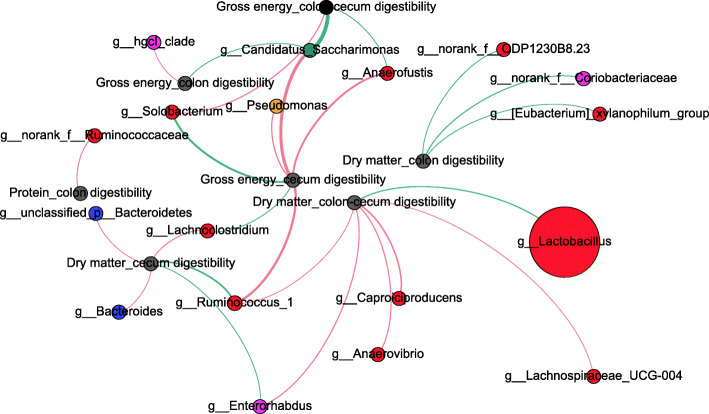
Correlation network analysis between nutrient digestibility and the microbiota of IUGR and NBW pigs. Networks display Spearman’s correlations between the relative abundance of bacterial genus and SCFAs concentrations. Only correlations with corrected *P* value < 0.05 are illustrated. Nodes are colored according to phylum, and the size is proportional to the mean relative abundance within the population. Blue edges represent negative correlation, while pink edges represent positive correlation. The thickness of edges is proportional to the correlation coefficient

The changing microbial composition may play a key role between undigestible nutrients and microbial fermentation in the large intestine. The *Firmicutes*, which were most dominant in IUGR pigs, are related to energy transformation [30]. We recently reported that IUGR piglets had a different gut bacterial community structure than NBW pigs during early-life [39]. At the genus level, *Lactobacillus* was most abundant bacterial genera in both the caecum and the colon of IUGR and NBW pigs, due to high percentage of Lactobacillus, the differential genera were less abundant, this predominant stuctrue damaged the microbial diversity and it has negative effects for stability of immune system. The caecum of IUGR pigs had higher levels of Ruminococcaceae and Lachnoclostridium*,* which are known for SCFA production. This is accord with the significantly high level of SCFAs concentration. Furthermore, *Lachnoclostridium*, *Enterorhabdus* and *Bacteroides* were positively correlated with dry matter digestibility. Lachnospiraceae were correlated with acetate and butyrate concentrations in the hindgut of NBW pigs (Fig. 7). The correlation analysis also showed that the Ruminococcaceae of the caecum and *Lactobacillus* of the colon were strongly correlated with DM digestibility (Fig. 8).

The present *in vivo* results further indicated that the differences in plasma metabolites between IUGR and NBW pigs may result mainly from the different rates of nutrient digestion in the ileum, thereby affecting fermentation in the caecum, hindgut microbiota. *In vitro* fermentation allowed us to study the causality between undigestible nutrients and microbial fermentation. The results indicated that the amount of the digesta leaving the small intestine was a key factor affecting microbial fermentation in the large intestine. The IUGR digesta inoculated with IUGR or NBW microbes both had a higher rate of gas production. Although the NBW digesta inoculated with NBW microbes had a higher rate of gas production than that with IUGR microbes, gas production was much lower in NBW than in IUGR digesta inoculated with IUGR or NBW, suggesting that the enhanced fermentation ability in the caecum of IUGR pigs was caused primarily by the undigested nutrients passing into the caecum. These data indicated that the whole fermentation process was influenced by both microbes and nutrients in digesta: the fermentation rate was influenced by caecal inocula and gas production was dependent on the ileal digesta.

In the swine industry, IUGR pigs are normally provided with more nutrients or high-density diets in an attempt to promote compensatory gain or catch-up growth. However, a previous study showed that a high nutrient specification diet in the grower phase does not improve the performance of IUGR pigs [40]. Our results indicated that the common commercial feeds already contained excessive levels of nutrients for IUGR pigs. These results indicate that effects must be made in future studies to improve the digestibility of nutrients in the small intestine and maintain a stable microbial community in the hindgut, rather than simply increase nutrient density in diets.

## Conclusion

This study investigated the characteristics of nutrient digestion, absorption, and hindgut fermentation in IUGR versus NBW pigs. Decreases in the digestibilities of DM, GE and starch measured at the ileal end compromised the nutritional status of IUGR pigs. The poor digestion in the small intestine provided the bacteria in the large intestine with abundant fermentable substrates such as starch, resulting in increased production of SCFAs in the caecum and reduced diversity of microbial community in the hindgut. These segmented differences in digestion and fermentation decreased the absorption of cholesterol, triglycerides and glucose into the portal vein, thus reducing the efficiency of nutrient utilization in IUGR pigs. Our results implied that nutrient digestibility in the small intestine and microbial fermentation in the hindgut should be fully considered to optimize feed efficiency for growth in IUGR offspring. These findings provided a new insight into understanding the nutritional defects of IUGR and a basis for future nutritional intervention through targeting different digestive sites.

## Data Availability

The datasets used and/or analysed during the current study are available from the corresponding author on request.

## Abbreviations

IUGR: Intrauterine growth restriction
NBW: Normal birth weight
ALB: Albumin
HDL: High-density lipoprotein
LDL: Low-density lipoprotein
DM: Dry matter
GE: Gross energy
SCFA: Short chain fatty acid
PAH: *p*-Amino hippurate
PVPF: Portal vein plasma flow
